# Supplementary material for: Working memory and reaction time variability mediate the relationship between polygenic risk and ADHD traits in a general population sample
Source: Mol Psychiatry. 2022 Sep 23;27(12):5028–37. doi: 10.1038/s41380-022-01775-5 (PMC9763105; doi:10.1038/s41380-022-01775-5)
Supplement: Supplementary file 1 — Supplementary figures [file 41380_2022_1775_MOESM1_ESM.pdf]

Working memory and reaction time variability mediate the relationship between polygenic risk and ADHD traits in a general population sample | **Supplementary figures**

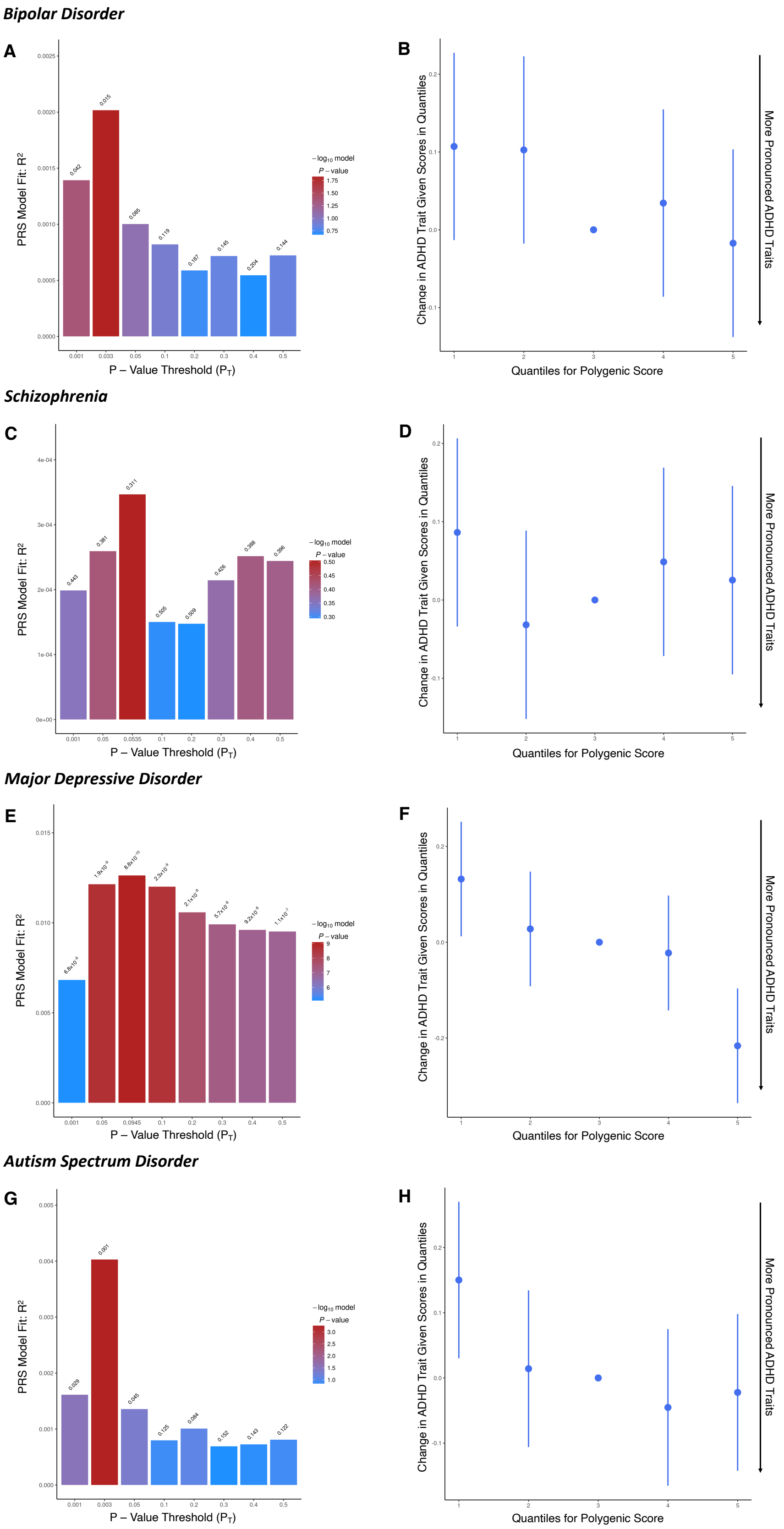

Supplementary Figure 1 **Associations between polygenic risk scores for bipolar disorder (A,B), schizophrenia (C,D), major depressive disorder (E,F), autism spectrum disorder (G,H) and ADHD trait scores.** First column in each subplot – bar plot showing the associations between ADHD trait scores and PRS across a range of p-value thresholds (p_T). Second column – quantile plot demonstrating the direction of the identified association at the best-fitting p_T. No significant associations were identified for bipolar disorder (A,B) and schizophrenia (C,D) PRS. PRS for major depressive disorder (p_T = 0.0945, R2 = 1.3%, p = 8.8 x 10^{-10}) and autism spectrum disorder (p_T = 0.003, R2 = 0.4%, p = 0.001) was associated with ADHD traits, where increased PRS was associated with increased ADHD traits (decreased factor scores). The total sample used in the PRS analyses, n = 2847. PRS = polygenic risk score.

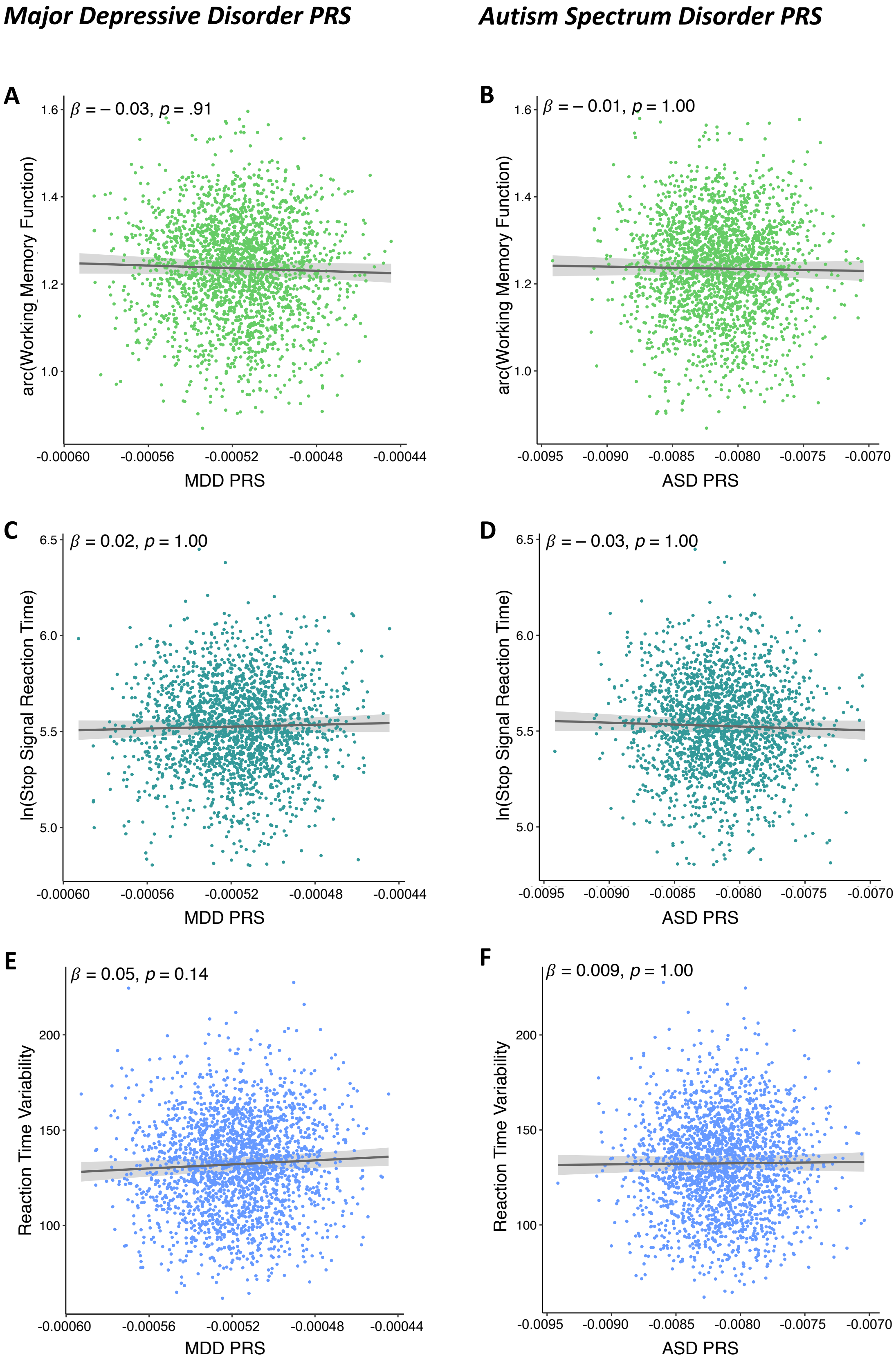

Supplementary Figure 2 **Associations between three ADHD candidate cognitive endophenotypes and MDD PRSs (column 1) and ASD PRSs (column 2).** No significant association was identified between MDD PRS and working memory accuracy scores form the emotional n-back task (β = – 0.03, p = 0.91, n = 2221, A); stop signal reaction time scores from the stop signal task (β = 0.02, p = 1.00, n = 2004, C); reaction time variability scores from the stop signal task (β = 0.05, p = 0.14, n = 2122, E) as well as between ASD PRS and PRS working memory accuracy scores (β = – 0.01, p = 1.00, n = 2221, B); stop signal reaction time scores (β = – 0.03, p = 1.00, n = 2004, D) and reaction time variability scores (β = 0.009, p = 1.00, n = 2122, F). All p-values are Bonferroni corrected for multiple comparisons. PRS = polygenic risk score; MDD = major depressive disorder; ASD = autism spectrum disorder.
